# High-Content Neurite Development Study Using Optically Patterned Substrates

**DOI:** 10.1371/journal.pone.0035911

**Published:** 2012-04-26

**Authors:** Jonathan M. Bélisle, Leonard A. Levin, Santiago Costantino

**Affiliations:** 1 Maisonneuve-Rosemont Hospital, University of Montreal, Montreal, Quebec, Canada; 2 Institute of Biomedical Engineering, University of Montreal, Montreal, Quebec, Canada; 3 Department of Ophthalmology, University of Montreal, Montreal, Quebec, Canada; Faculty of Medicine University of Leipzig, Germany

## Abstract

The study of neurite guidance *in vitro* relies on the ability to reproduce the distribution of attractive and repulsive guidance molecules normally expressed *in vivo*. The identification of subtle variations in the neurite response to changes in the spatial distribution of extracellular molecules can be achieved by monitoring the behavior of cells on protein gradients. To do this, automated high-content screening assays are needed to quantify the morphological changes resulting from growth on gradients of guidance molecules. Here, we present the use of laser-assisted protein adsorption by photobleaching (LAPAP) to allow the fabrication of large-scale substrate-bound laminin-1 gradients to study neurite extension. We produced thousands of gradients of different slopes and analyzed the variations in neurite attraction of neuron-like cells (RGC-5). An image analysis algorithm processed bright field microscopy images, detecting each cell and quantifying the soma centroid and the initiation, terminal and turning angles of the longest neurite.

## Introduction

During embryogenesis, neurons extend their axons over long distances in order to form the complex circuitry of the central nervous system. At the tip of the axon, a specialized dynamic structure called the growth cone senses the distribution of specific proteins and in response, modifies its shape to guide such extension [1], [2], [3]. This reshaping process is based on the response to several molecules that act as guidance cues. These trigger specific signaling pathways to locally reorganize microtubules and actin filaments that appropriately steer the growth cone. Several *in vitro* methods have been developed to mimic the spatial distributions of proteins that are found in vivo [4], [5], [6] for studying the growth cone. Micropipette puffed generated gradients [7], [8] have been extensively used and help to better understand the molecular mechanisms of axonal guidance. However, this technique allows study of only one axon at a time, and usually 10–50 cells per condition in total are analyzed [8], [9], [10], [11], [12]. Even with methods that allow study of multiple cells in parallel, such as microfluidic generated gradients [13], [14], the number of neurons analyzed per condition is often relatively low, making it difficult to identify subtle variations in axonal guidance. Studying a few neurons per condition is helpful when axons turn *en masse* in response to a molecular gradient, but when only a fraction of the cell population responds, thousands of neurons may be necessary to understand the biological basis [15].

To overcome the limitation on the number of cells analyzed, high-content screening assays have recently been developed to obtain information about large populations of neurons. High-content analysis combines automated or semi-automated microscopy that obtain a large number of images with detailed image analysis to collect quantitative and standardized information about cell responses, e.g. morphology and protein expression [16]. High-content analysis has already been applied successfully in drug discovery [17], [18], RNAi screening [19], [20], and most recently to the study of neurite outgrowth [21], [22], [23]. Automated analysis of images using morphology filters allows extraction of particular features for multiple neurons. These include information about their somas, axons, and dendrites, as well as outgrowth and branching information for thousands of cells, permitting quantitative comparison of morphologies in different conditions.

The ability to fabricate several identical protein patterns is a prerequisite for performing high-content analysis of axonal guidance. To achieve this, we recently developed a technique for fabricating substrate-bound protein patterns, which allows the production of hundreds of patterns in parallel. Laser-assisted protein adsorption by photobleaching (LAPAP) [24], [25] uses a laser to photobleach fluorophores conjugated to various molecules as the basis for producing protein patterns, where the intensity of the laser modulates the final concentration of protein bound. Typically, biotin-4-fluorescein is used as the first binding molecule, followed sequentially by binding of streptavidin, biotinylated antibodies, and the protein of interest. Originally, LAPAP was able to produce protein distributions with micrometer accuracy using a high numerical aperture objective lens, at the cost of relatively slow fabrication. For fabricating gradients more rapidly, a simple lens can be used to produce low-resolution protein patterns at approximately a minute per pattern.

Early work on neurons from the sympathetic ganglia of chick embryos did not show any evidence of influence from a substrate-bound laminin gradient upon the orientation of axons [26]. However, subsequent experiments showed that DRGs from chick embryos were turning on substrate-bound gradients from a laminin peptide, IKVAK [27]. It was shown that laminin gradients were influencing the direction of the presumptive axon (longest neurite) of rat hippocampal neurons [13] or neurites from PC12 cells [28], as well as to induce turning in axons from chick retinal explant [29] or *Xenopus* spinal neurons [30]. *In vivo* experiments with laminin zebrafish mutants showed axons from various neural types made specific pathfinding errors [31], therefore suggesting that laminin is implicated in guidance. Recently, it has been shown that laminin triggers a new signaling pathway for neuritogenesis [32] as well as to influence retinal ganglion cells (RGC) polarisation for the appropriate orientation of axon emergence [33]. Since laminin is not considered a strong attracting protein but has shown certain attracting effect, it became a good candidate for high-content analysis in the hope of unraveling small neurite growth effects. Here we present the use of large-scale low-resolution LAPAP to produce 1350 gradients for high-content screening of a neuronal cell line (RGC-5) to demonstrate in a reliable manner the effect of laminin gradients on the orientation of neurites. RGC-5 cells were derived from developing rodent retina; they differentiate and exhibit neuronal morphology when treated with low concentrations of staurosporine [34]. This cell line represents a good model for testing high-content analysis combined with substrate-bound protein patterning since RGC-5s express various neurotrophins as well as their receptors and have a similar phenotype compared to RGCs [35]. RGC-5 cells were sequentially imaged over a 4 h period before and after differentiation with staurosporine. Approximately 7000 bright-field images of neurons on laminin-1 gradients and control patterns were taken in a semi-automated manner and analyzed using a fully automated image processing algorithm. Quantitative measures of cell morphology were obtained, e.g. position of the soma centroid, most distal neurite point, longest neurite length, and neurite initiation, terminal, and turning angles.

## Materials and Methods

### Laser protein patterning

**Figure 1:**
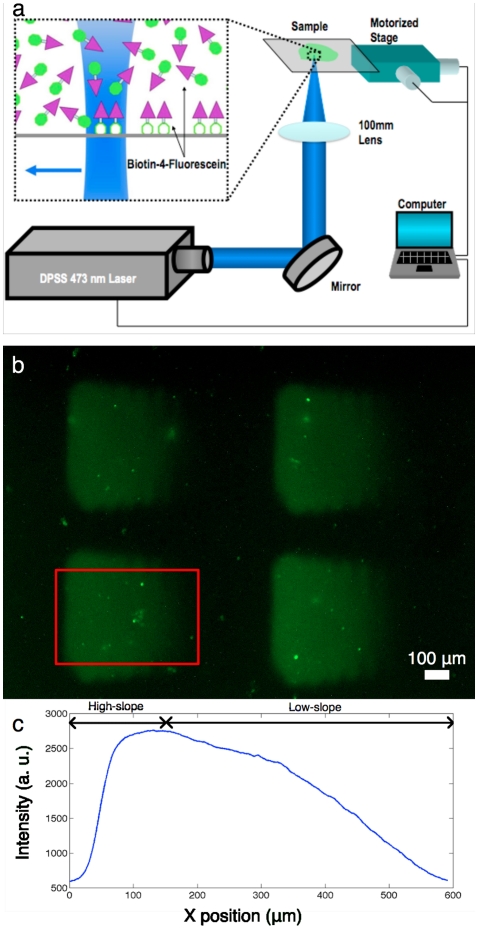
Patterns of laminin-1 produced using LAPAP. (a) The platform consists of a 473 nm diode-pumped solid-state laser, a lens and a 3-axis motorized stage to produce the desired patterns of biotin-4-fluorescein. (b) Large gradients (400 µm×600 µm) of laminin-1 were fabricated by sequentially incubating streptavidin-DyLight549, biotinylated goat anti-rabbit IgG (Fc specific), rabbit anti-laminin-1 and laminin-1. This particular pattern was imaged using chicken anti-laminin-1 and Cy5 goat anti-chicken IgG to reveal the presence of laminin-1. The red rectangle delimits the section of the patterns considered for the RGC-5 guidance assay. (c) x-direction mean profile of 25 gradients. The first quartile (on the left) of the pattern was used as a high-slope gradient while the last three quartiles (on the right) were used as a low-slope gradient.

The laser protein patterning method has previously been described in detail [24]. Briefly, the LAPAP setup (Fig. 1a) consists of a 473 nm diode-pumped solid-state laser (Laserglow, ON, Canada) focused by a 100 mm plano-convex lens. The sample is moved with respect to the laser focal spot by a 3-axis translation stage (Thorlabs, NJ), with the laser power and translation stage positions controlled by a custom-written Labview program (National Instrument, TX).

A glass bottom dish (MatTek Corp., MA) was incubated with 3% bovine serum albumin (BSA) in PBS for 30 minutes at room temperature, followed by biotin-4-fluorescein (50 µg/ml in 3% BSA). The laser scanned the sample at a speed of 150 µm/s, resulting in binding of the biotin-4-fluorescein to the glass. Fifteen equally spaced lines 400 µm long by 40 µm were patterned as the laser power was increased from 0.02 mW to 4 mW. The width of each line was approximately 100 µm, which yielded patterns with a continuous graded intensity. This pattern size corresponds to the field of view obtained on the CCD when imaging with a 20× objective, and is therefore optimal for later imaging of the cells extending their neurites on the gradient. To produce gradients of laminin-1, we subsequently incubated streptavidin-Cy5 (Jackson ImmunoResearch, PA), biotinylated goat anti-rabbit Fc-specific IgG (Jackson ImmunoResearch, PA), polyclonal rabbit anti-laminin IgG (L9393, Sigma, MO), and laminin-1 from murine sarcoma basement membrane (Sigma, MO), each at 5 µg/ml in 3% BSA for 30 minutes at room temperature. The use of Fc-specific goat anti-rabbit IgG is necessary to bind the rabbit anti-laminin by its Fc region in order for the next antibody to have an orientation that facilitates laminin-1 binding. Moreover, a polyclonal rabbit anti-laminin was used to expose laminin molecules bound to the surface with several different orientations, thus avoiding blocking the interaction with cellular receptors. Between each incubation step, the patterns were rinsed 4–5 times with phosphate buffered saline (PBS). To confirm the presence of laminin-1, we fabricated patterns where streptavidin-Cy5 was replaced by streptavidin-DyLight549 and added two supplementary incubation steps, chicken anti-laminin (5 µg/ml in 3% BSA) and Cy5 goat anti-chicken (5 µg/ml in 3% BSA), after laminin-1 incubation. Fig. 1b shows the fluorescence from Cy5 goat anti-chicken of this particular pattern and the red rectangle shows the region that is considered for neurite guidance. Fig. 1c shows the mean profile of 25 gradients where the first quartile (left) was used as a high-slope gradient and the last three quartiles (right) as a low-slope gradient. On average the change in protein concentration in the high-slope gradients is three times steeper than in low-slope ones. We designed the gradients to be linear (Fig. 1c) in order to study constant concentration changes of laminin [36]. Considering a 20 µm cell diameter, the absolute concentration change on each side of the cell for high-slope gradients is 13.9% of the maximum laminin concentration and for low-slope gradients is 4.6%. Control gradients were also patterned where all steps were performed up to rabbit anti-laminin incubation, and are depicted as control 1. Fifty gradients (25 low-slopes and 25 high-slopes) were patterned per culture dish, totalling 800 laminin-1 gradients and 550 anti-laminin gradients for control 1.

### Cell culture and differentiation

RGC-5 cells (kind gift of Neeraj Agarwal, PhD) were cultured in Dulbecco's modified Eagle medium (DMEM) supplemented with 10% fetal bovine serum, 100 U/mL penicillin, and 100 µg/mL streptomycin. Cells were plated on glass-bottom dishes containing laminin-1 patterns, anti-laminin patterns (control 1) or no patterns (control 2) at a density of 1000 cells/cm^2^ and incubated overnight at 37°C in 5% CO_2_. RGC-5 cells were then differentiated the next morning by replacing culture media with staurosporine (316 nM).

### Microscopy

Fluorescence images of gradients and bright field images of cells were acquired with an inverted microscope (IX71 Olympus, Japan) equipped with a Retiga 2000R CCD camera (QImaging, Canada). The gain, exposure time and the other parameters of the CCD were identical for all image acquisitions. For samples with gradients, the culture dishes were first positioned manually by observing the fluorescence of streptavidin-Cy5 to image the protein patterns. The sample was rotated so that we positioned the high-slope gradient increasing horizontally from left to right and the low-slope gradient from right to left. Then, patterns were imaged in fluorescence mode and the RGC-5 cells in bright field mode using a 20× 0.75NA objective. Each image contained both the low-slope and high-slope gradients, and was split in two for analysis. Images corresponding to high-slope gradients with cells on them were horizontally flipped to always have gradients with increasing concentration from right to left. For control samples with no gradients, semi-automated (fine focus adjustment was controlled manually) bright field microscopy was performed using a motorized translation stage (Thorlabs, NJ) controlled by a custom program written in Labview (National Instrument, TX). During each imaging session, cells were at room temperature and exposed to room air for less than 10 minutes, and were then placed back in the incubator at 37°C in 5% CO_2_.

### Automated image analysis

This section describes an algorithm that combines basic image processing operations that are explained in detail elsewhere [37]. Image analysis was performed using an algorithm written in MATLAB™ (Mathworks, MA) and made use of built-in functions of the Image Processing Toolbox™ similar to what has been presented previously [38], [39]. All parameters that were manually set by the user for the image analysis were kept constant for all samples. The MATLAB code is available upon request.

Bright field images were first normalized, and then a Sobel filter was performed to find the edges of the cells. From the resulting binary image, isolated pixels were removed. The image was then dilated by 3 pixels in order to avoid breaks in neurites, and holes were filled to obtain masks for all objects in the image. Only objects with masks sized 685–4107 µm^2^ (5000–30000 pixels) were considered neurons, with smaller objects removed.

Using these masks of individual neurons, a morphological opening was performed using a disk of 11.1 µm (30 pixels) diameter as a structuring element in order to create a mask of the soma. Objects with soma masks smaller than 274 µm^2^ (2000 pixels) or larger than 1369 µm^2^ (10000 pixels) were discarded. Neuron masks where no soma or more than one was detected were also removed from the analysis. For those containing only one soma, the centroid of the soma was detected using the function *regionprops* (*‘centroid’*).

To find the skeleton of a neuron, a thinning of the neuron mask was performed using the function *bwmorph* (‘thin’) until minimally connected lines were obtained. The locations of the skeleton end-points and intersection-points were then obtained using the algorithms *find_skel_ends* and *find_skel_intersection* available in MATLAB Central (http://www.mathworks.com/matlabcentral/).

**Figure 2:**
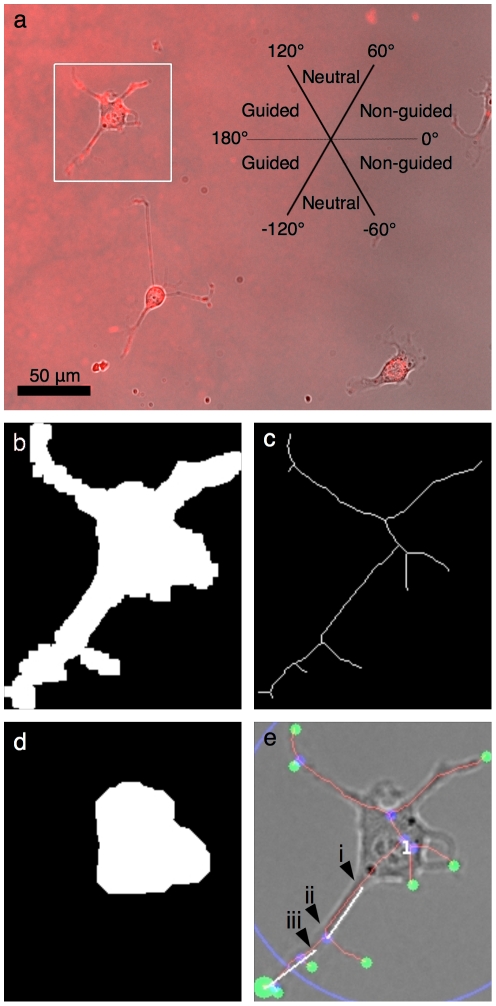
Image analysis algorithm key steps. RGC-5 cells on a low-slope laminin-1 gradient where a streptavidin-Cy5 gradient was imaged by fluorescence microscopy with cells in bright field mode using a 20× 0.75 NA objective. (a) The properties of the longest neurite of each cell were classified as attracted, neutral or repulsed. (b–e) In order to study thousands of cells, classify them in an objective manner and collect information about each cell, an automated analysis algorithm was written in MATLAB™. Key steps of the algorithm are depicted here for the cell denoted by the white square in panel (a). (b) First, a Sobel filter, dilation, elimination of small objects and filling of remaining object was used to define a mask for each cell. (c) A skeleton was computed by performing a thinning of the mask corresponding to each cell until minimally connected lines were obtained. (d) A morphological opening of the mask was then performed to find the region corresponding to the soma and then its centroid (marked by the upper left position of the number in panel e). (e) The position of all end-points (green) and intersection-points (blue) of the skeleton were determined. The position of the most distal end-point (large green disk) relative to the soma's centroid and the length of the corresponding skeleton branch were computed, as well as the initiation angle and the terminal angle.

The most distal skeleton end-point from the soma's centroid was determined and further analysis was performed on the skeleton branch associated with that end-point. To find the initiation angle, the position where that single branch intersected the soma mask was determined (Fig. 2e, arrow i) as the initiating location of the neurite. The position on the skeleton branch 16.7 µm (45 pixels) away from the initiating point was then found (Fig. 2e, arrow ii) and a straight line between those two positions was traced to determine the initiation angle. A value of 16.7 µm (45 pixels) was chosen since it corresponds to the median value of the soma radius calculated from all the analyzed cells. A 0° angle corresponds to a neurite initiating extension in the direction of decreasing slope (left to right) of the gradient and a ±180° angle to a neurite extending in the direction of increasing slope (right to left). Positive values were assigned to neurites initiating upwards and a negative value to neurites initiating downwards (Fig. 2a). The angle of neurite termination was determined from the segment between the most distal end-point from the soma centroid (Fig. 2e, big green disk) and the point 16.7 µm (45 pixels) away on the same skeleton branch (Fig. 2e, arrow iii). Branches smaller than 45 pixels in length were not analyzed.

## Results and Discussion

The morphological parameters obtained by automated analysis allowed us to observe the impact of laminin-1 gradients on neurite outgrowth. Subtle influences of the gradient could be measured and the collection of several parameters allowed us to better assess a variety of effects on neurite outgrowth.

**Figure 3:**
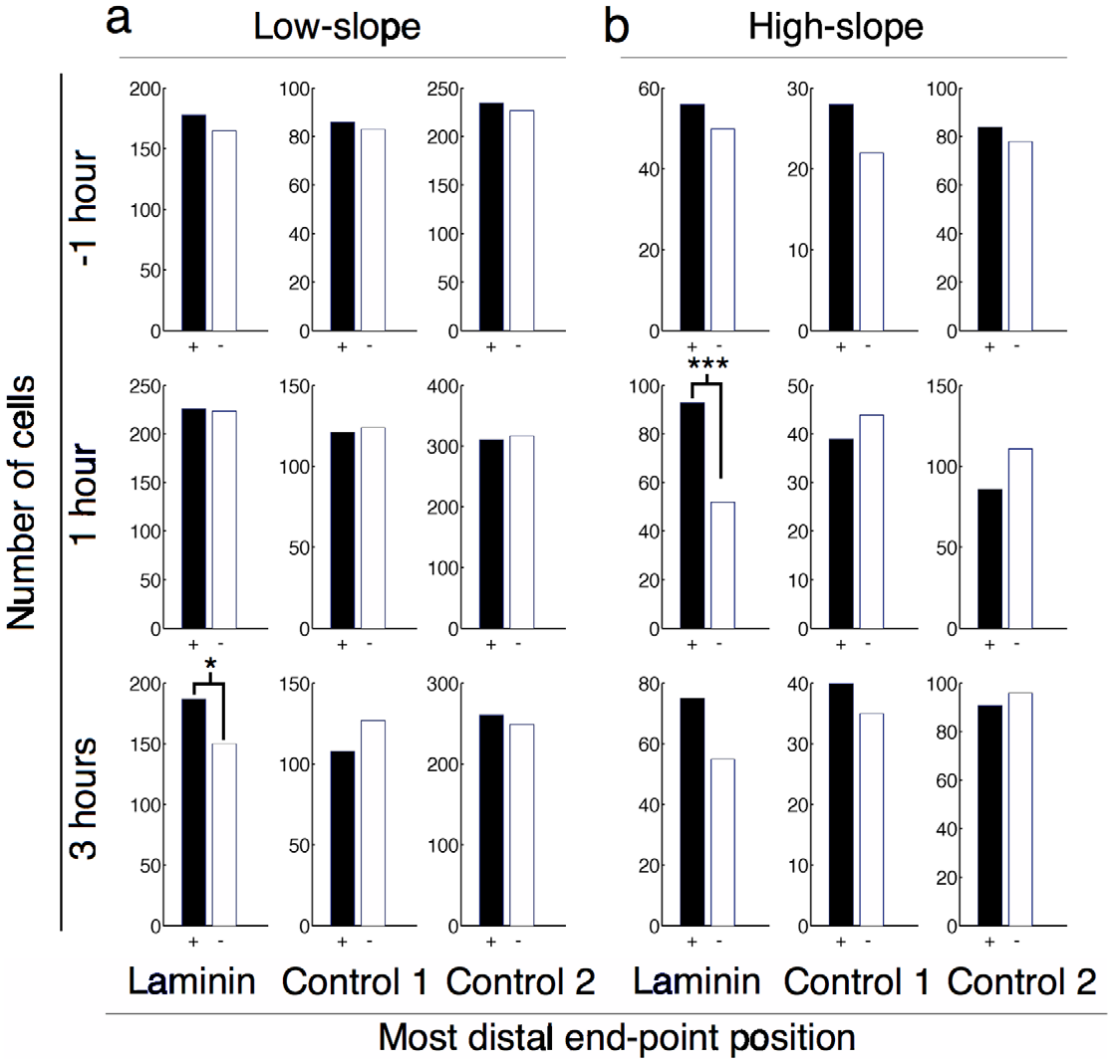
Neurite extension bias according to most distal end-point. Histograms of most distal end-point relative to soma centroid position for all cell images in both low-slope and high-slope gradients at time points before and during differentiation with staurosporine. Attracted (black, +) and repulsed (white, −) neurites were determined as described in Figure 2a. Low-slope gradients resulted in statistically significant guidance for laminin-1 three hours after differentiation (p = 0.044 by χ^2^). High-slope gradients resulted in guidance that was statistically significant at 1 hour (p = 0.0006 by χ^2^) after differentiation by staurosporine.

Fig. 3 shows histograms of the location of the most distal end-point of the longest neurite compared to the position of the centroid of the soma. Attracted neurites (black, +) are those extending in the direction of the gradient as described by the inset in Fig. 2a. Repulsed neurites (white, −) extend in the opposite direction and neutral neurites (not shown) are those extending perpendicular to the slope direction. Fig. 3 shows the data corresponding to a total of 4514 cells analyzed (7100 if we include neutral cells).

When cells extended neurites on high-slope gradients (Fig. 3b), we observed statistically significant guidance (p = 0.0006, χ^2^ test) 1 hour after differentiation for the most distal end-point data with 93 cells marked as attracted and 52 marked as repulsed. This result raised other questions: Does the laminin-1 gradient increase the length of neurite outgrowth in the positive slope direction? Are neurites more likely to be initiated with an angle corresponding to the direction of the gradient, or is the neurite turning along the gradient during its extension?

**Figure 4:**
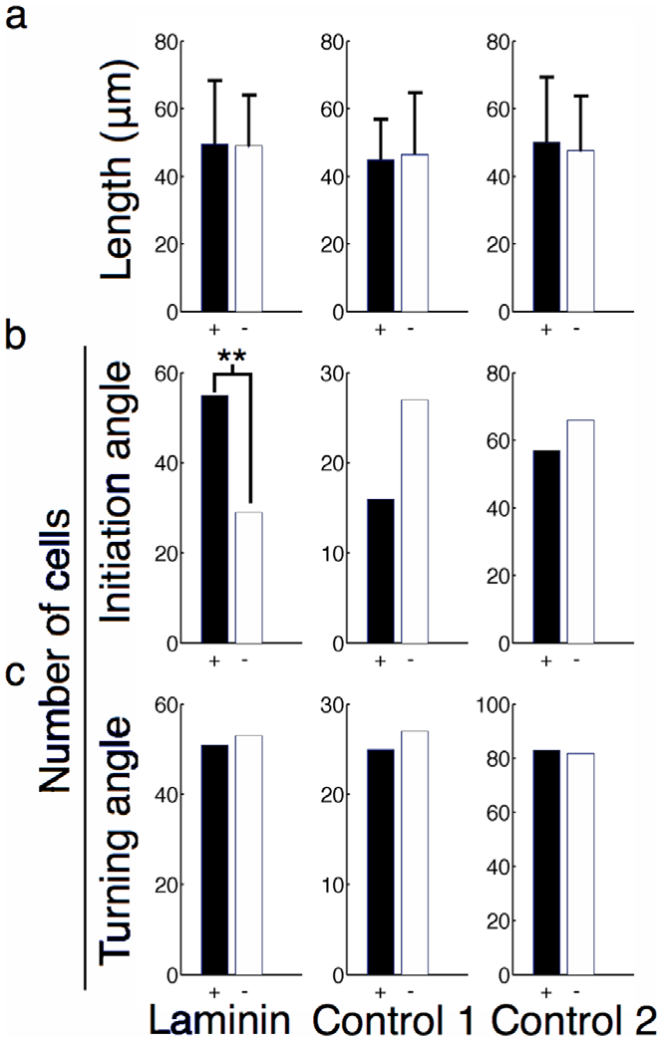
Various parameters of cells extending neurites on high-slope gradients 1 hour after differentiation. (a) The length of the neurite associated with the most distant end-point was similar for attracted and repulsed cells (p = 0.798 by t-test). (b) The histogram of the initiation angles of the neurites indicates that the angle at which they sprouted from the soma was responsible for the guidance (p = 0.005 by χ^2^). (c) The histogram of the turning angle shows that turning while the neurite was extending was not responsible for guidance.

The average neurite length, calculated as the distance from initiation point (Fig. 2e, arrow i) to the maximum end-point, for attracted, neutral, and repulsed neurites is shown in Fig. 4a for high-slope gradients 1 hour after differentiation. No statistically significant difference could be observed for this parameter. We then compared the initiation angle (Fig. 4b) of the longest neurite and saw an influence of the laminin-1 gradient that was statistically significant (p = 0.005, χ^2^ test) with 55 cells considered attracted and 29 repulsed. Fig. 4c shows the turning angle (towards the gradient), defined as the difference between the absolute value of the terminal angle and initiation angle. A positive turning angle means that a neurite turned toward the increasing slope of the gradient. The histogram in Fig. 4c considers a turning angle greater then 5° as being attracted (black, +), one between −5° and 5° as neutral (not shown) and one smaller than −5° as being repulsed (white, −). Again, no statistically significant turning could be observed, therefore establishing that only the initiation of the neurite is influenced by the substrate bound gradient. The complete data obtained for all time points is presented in Figures S1, S2, S3, S4, S5, S6.

As shown in Fig. 3a, low-slope laminin gradients influence neurite outgrowth and is statistically significant 3 hours after differentiation guidance with 187 attracted cells and 150 repulsed (p = 0.044, χ^2^ test). Initiation, terminal, and turning angles data were not significantly influenced (see Fig. S1a–S3a) by low-slope laminin gradients. In addition, the distribution of cell somas along the gradient was not significantly affected by high or low concentrations of laminin (see Fig. S4), nor was neurite branching (see Fig. S5a).

**Figure 5:**
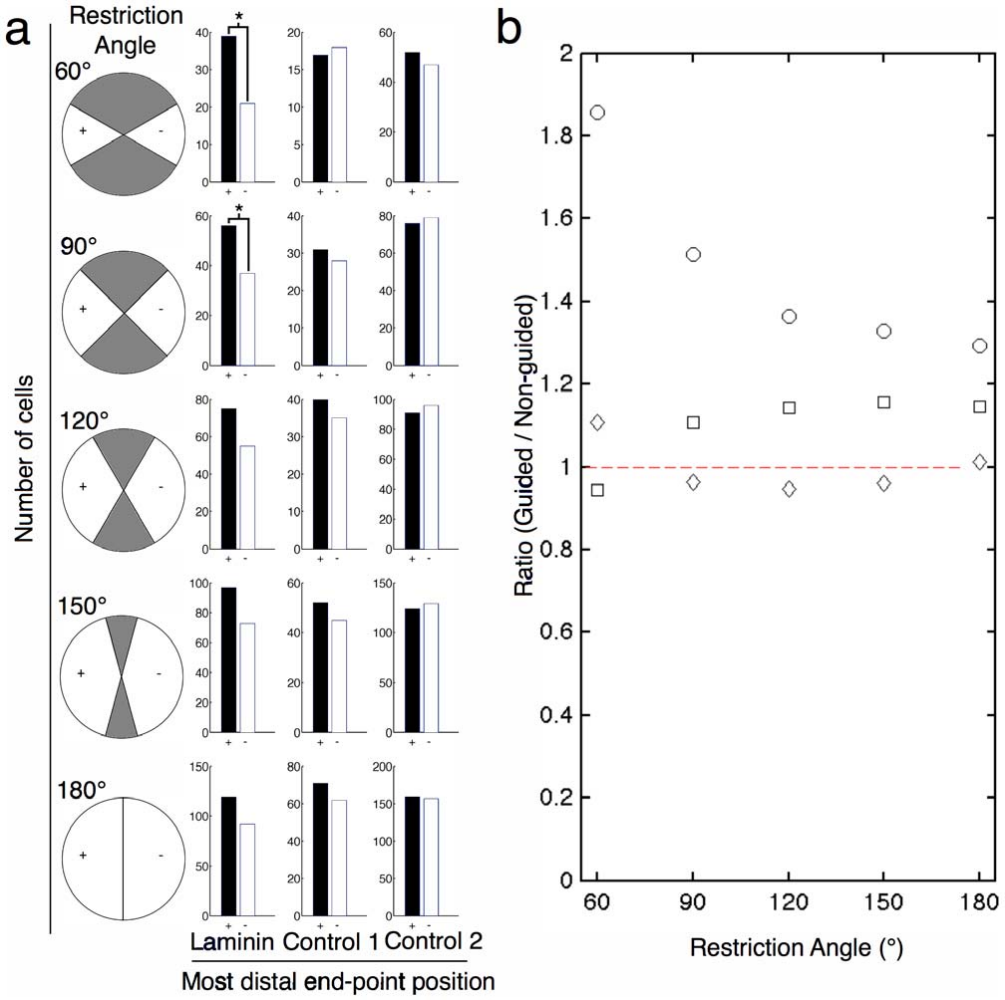
Effect of varying the restriction angle used to define whether an extending neurite was attracted or repusled by the gradient. (a) Histograms of the most distal end-point position for different restriction angle. (b) Ratio of attracted over repulsed neurites shows how this arbitrary parameter can greatly influence the outcome of the analysis. (Circles = Laminin, Squares = Control 1, Diamonds = Control 2). This analysis demonstrates that high-content screens of neurite guidance are strongly dependent on choice of critical user-defined parameters. Data used from high-slope gradients, three hours after differentiation.

The angular range for determining guidance was arbitrarily set at 120° for each possibility (see Fig. 2a): a 120° angle in the direction of the increasing slope (left) of the gradient was defined as attracted, a 120° angle in the opposite direction (right) was defined as repulsed and the two 60° angles perpendicular (up and down) to the gradient was defined as neutral. In Fig. 5, we analyzed a data set (high-slope gradients 3 hours after differentiation) that did not show significant guidance (see Fig. 3b). The ranges of the angle were varied from 60° to 180° to show how the angular range influenced the ratio of attracted to repulsed neurites (Fig. 5). By restricting the angular range, we observed statistically significant guidance for restriction angles of 60° and 90° (Fig. 5a). This result emphasizes the importance of establishing standards for user-defined parameters for high-content analysis of neurite guidance.

Even if axons from chick embryos retinal explant have already been shown to turn on laminin gradients [29], here we only report bias in neurite initial growth rather then reorientation for mammalian RGCs. Moreover, for high slope gradients, the influence in initial orientation is transient, which is consistent with the fact that mammalian RGCs lose affinity with laminin with maturation [40]. The mechanisms that influence neurite initiation are beyond the scope of our study, however we set the methodological basis for high-content screens that can lead to these responses. In particular, neuritogenesis signalling pathways that are dependent on laminin for actin meshwork reorganization [32].

Moreover, several morphological changes could not be assessed correctly with our system due to the lack of temporal resolution. For example, the bias seen on high slope laminin gradients 1 hour after differentiation is not present at 3 hours (Fig. 3b). High-content microscopy of living cells over several hours would allow tracking intermediate changes occurring with a higher temporal resolution.

### Conclusion

We used protein patterns produced by LAPAP for high-content screens to study neurite guidance. The image analysis tools developed for this study made possible automated neurite tracing from bright field images on substrate bound gradients. Previous studies of automated quantification of neurite outgrowth typically used fluorescence microscopy images because they allowed simpler neurite detection, necessitating stained cells or live cells expressing genetically encoded fluorescent probes [22], [38]. The morphological parameters obtained from the automated analysis allowed a quantitative understanding of the preferential neurite outgrowth of RGC-5 on laminin-1 gradients.

We showed that such preferential neurite guidance could be observed by considering the position of the most distal end-point of the longest neurite relative to the soma on low and high-slopes laminin-1 gradients. On average, the longest neurites of cells had similar lengths, independently of their orientation with respect to the protein gradient. For high-slope laminin gradients, we also demonstrated that the guidance observed was mainly due to the initiation angle of neurites, which was preferentially in the direction of the increasing concentration of the gradient, and not turning of the longest neurite as it extended on the gradient. These finding are consistent with fact that neuritogenesis can occur through a different signaling pathway in the presence of laminin [32], therefore having neurites more likely to initiate where laminin is of higher concentration.

The work presented here is a proof of concept for the application of high-content analysis to study neurite guidance on substrate-bound protein patterns. The use of LAPAP to produce multiple protein distributions and automated image analysis of the observed cells allows observation of large numbers of cells to tease out weak guidance cues, and is a first step towards a totally automated platform combining LAPAP, cell culture and microscopy for the screening of substrate-bound guidance cues in different types of neurons.

## Supporting Information

Figure S1 Histograms of the initiation angle of cells imaged on low-slope and high-slope gradients at different time points before and during differentiation by staurosporine. Attracted (black, +) and repulsed (white, −) neurites were determined as described in Figure 2a.
(TIF)

Figure S2 Histograms of the terminal angle of cells imaged on low-slope and high-slope gradients at different time points before and during differentiation by staurosporine. Attracted (black, +) and repulsed (white, −) neurites were determined as described in Figure 2a.
(TIF)

Figure S3 Histograms of the turning angle of cells imaged on low-slope and high-slope gradients at different time points before and during differentiation by staurosporine. Attracted (black, +) and repulsed (white, −) neurites were determined as described in Figure 2a.
(TIF)

Figure S4 Histograms of the x-position of the centroid of the cell soma along the gradient (low-slope gradient). The laminin-1 or anti-laminin (control 1) concentration was highest at 0 µm and lowest at 444 µm.
(TIF)

Figure S5 Histograms of the branching points of cells imaged on low-slope and high-slope gradients at different time points before and during differentiation by staurosporine. Attracted (black, +) and repulsed (white, −) neurites were determined as described in Figure 2a.
(TIF)

Figure S6 Histograms of the x-position of the centroid of the cell soma along the gradients (high-slope gradient). The laminin-1 or anti-laminin (control 1) concentration was highest at 0 µm and lowest at 148 µm.
(TIF)
