# Supplementary figures and images for: High-Content Neurite Development Study Using Optically Patterned Substrates
Source: PLoS One. 2012 Apr 26;7(4):e35911. doi: 10.1371/journal.pone.0035911 (PMC3338543; doi:10.1371/journal.pone.0035911)

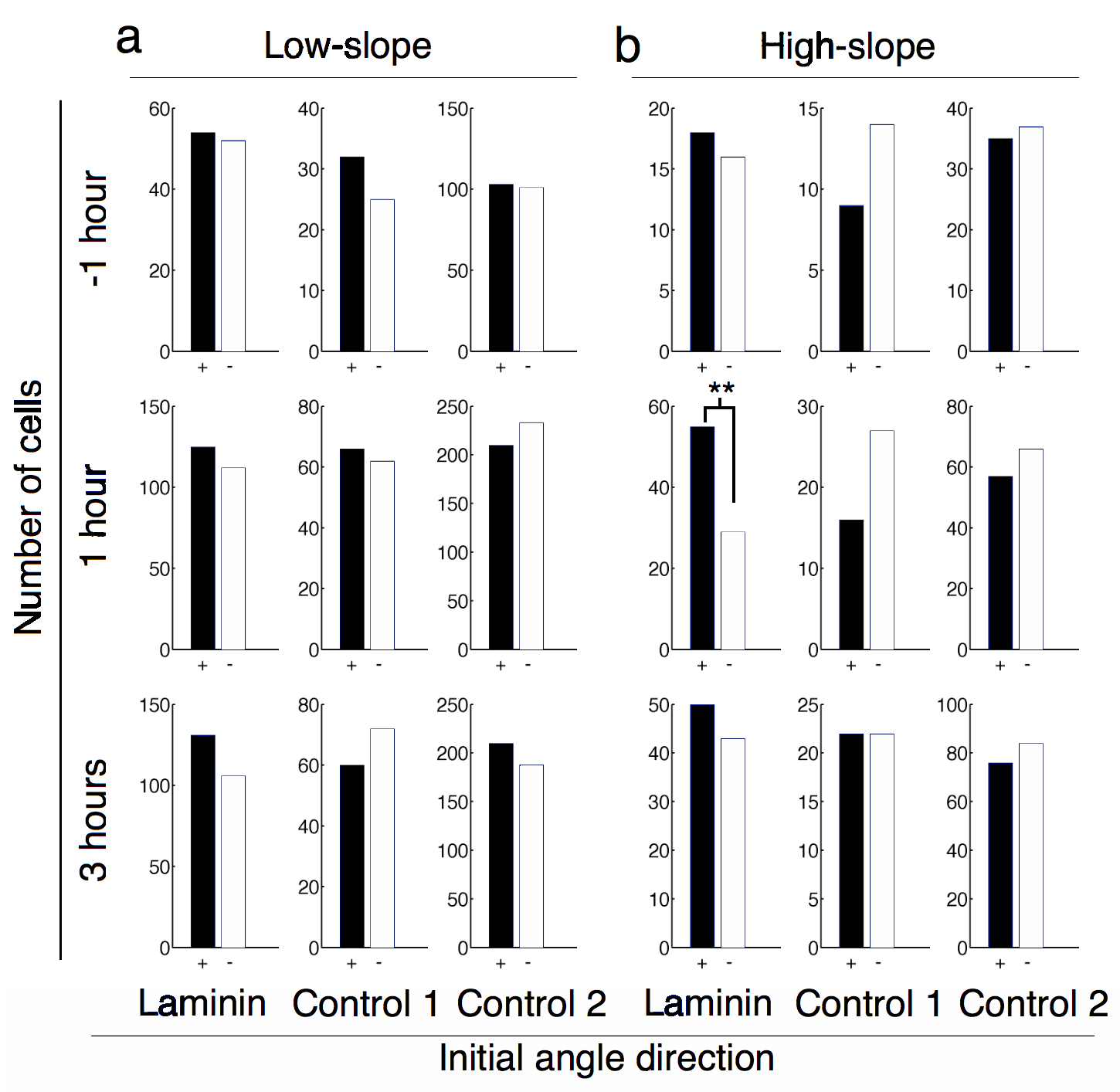

Supplement: Figure S1 — Histograms of the initiation angle of cells imaged on low-slope and high-slope gradients at different time points before and during differentiation by staurosporine. Attracted (black, +) and repulsed (white, −) neurites were determined as described in Figure 2a. (TIF) [file pone.0035911.s001.tif]

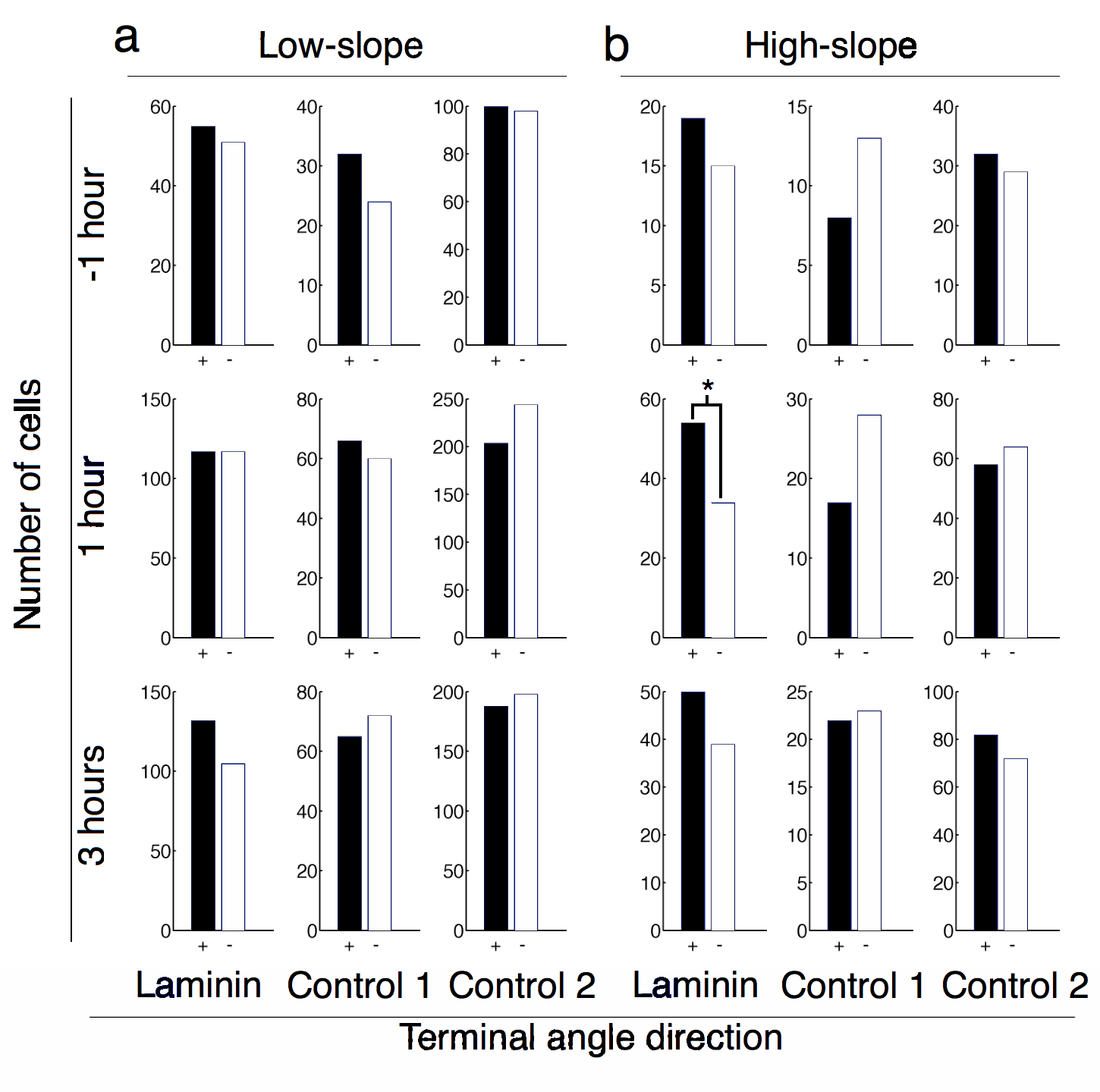

Supplement: Figure S2 — Histograms of the terminal angle of cells imaged on low-slope and high-slope gradients at different time points before and during differentiation by staurosporine. Attracted (black, +) and repulsed (white, −) neurites were determined as described in Figure 2a. (TIF) [file pone.0035911.s002.tif]

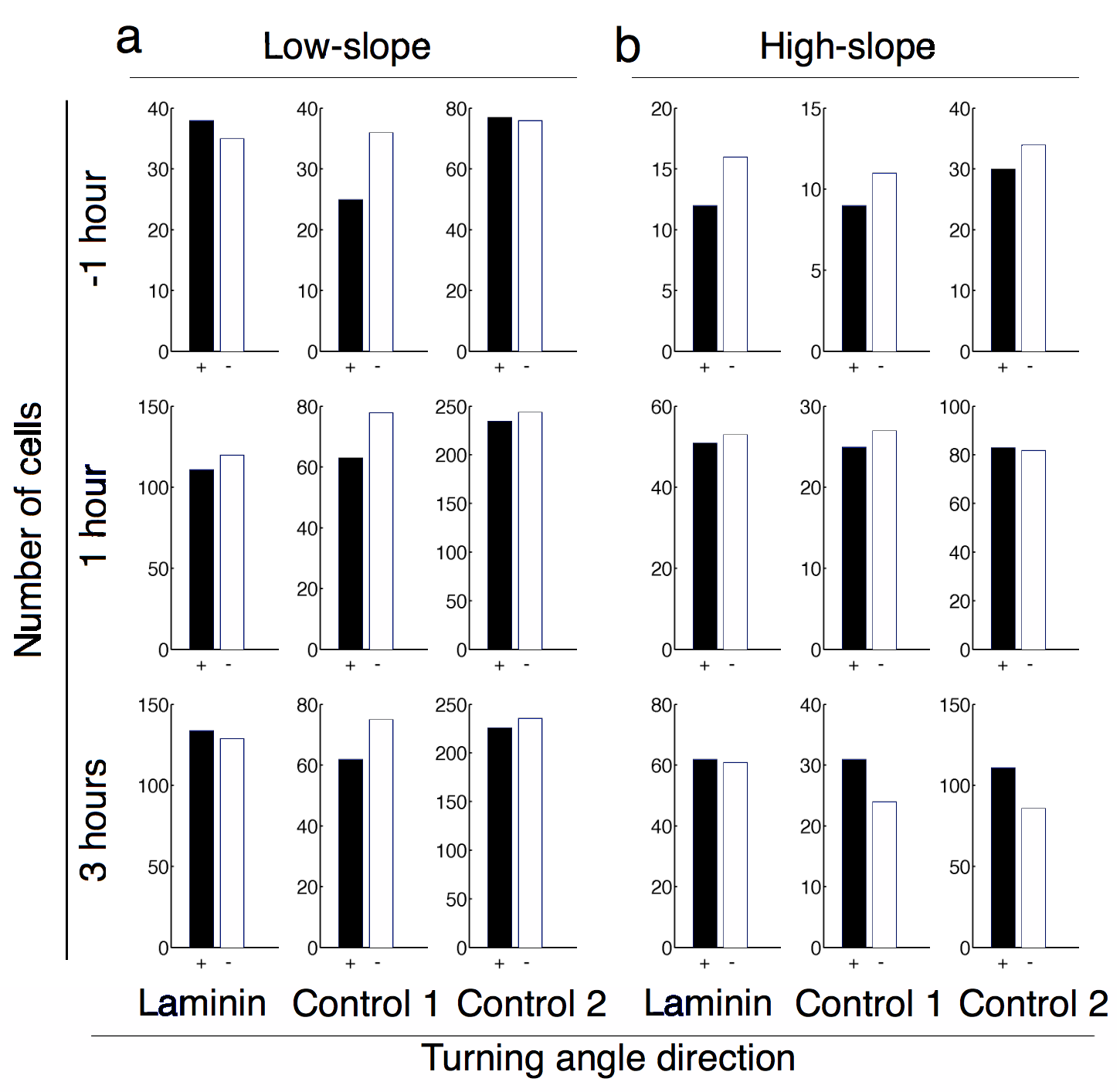

Supplement: Figure S3 — Histograms of the turning angle of cells imaged on low-slope and high-slope gradients at different time points before and during differentiation by staurosporine. Attracted (black, +) and repulsed (white, −) neurites were determined as described in Figure 2a. (TIF) [file pone.0035911.s003.tif]

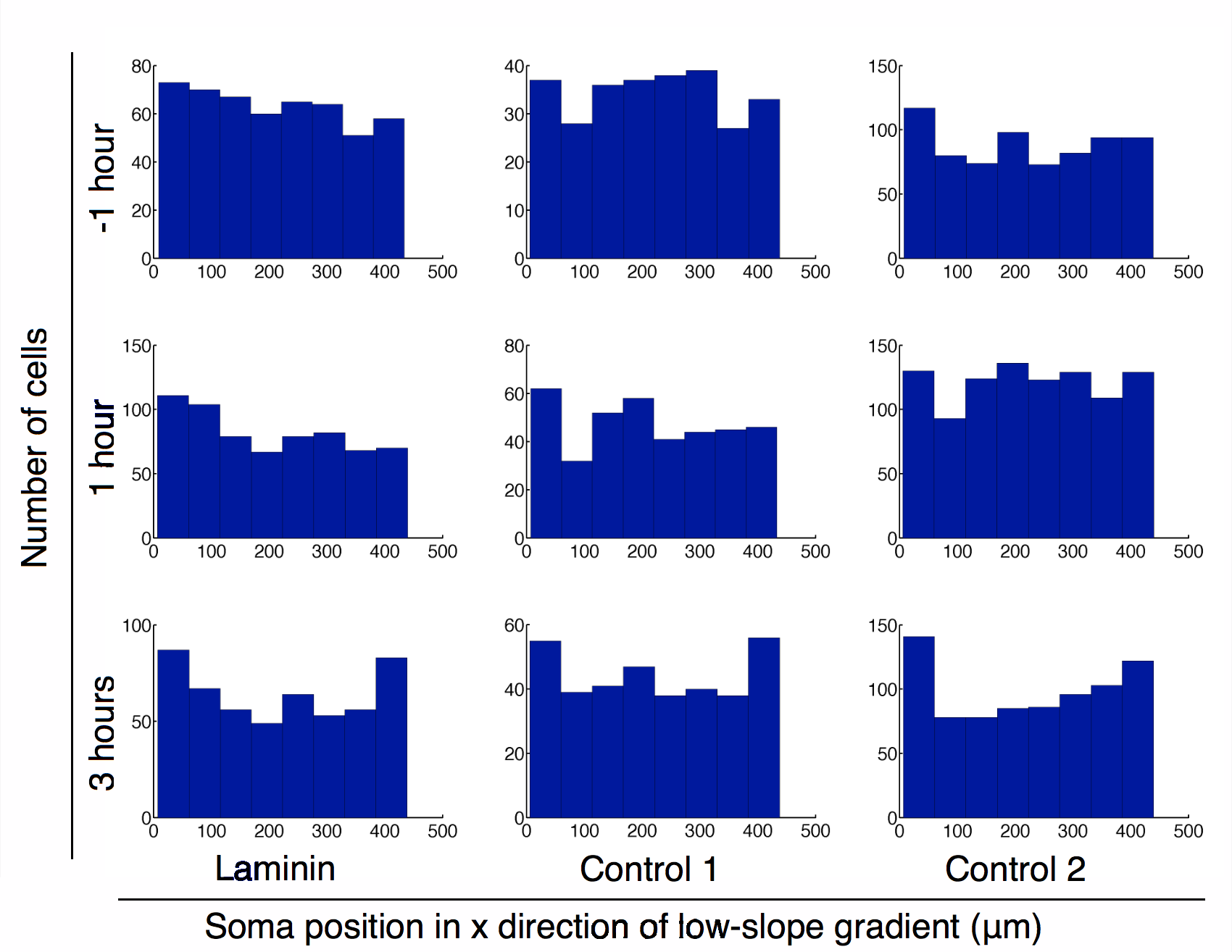

Supplement: Figure S4 — Histograms of the x-position of the centroid of the cell soma along the gradient (low-slope gradient). The laminin-1 or anti-laminin (control 1) concentration was highest at 0 µm and lowest at 444 µm. (TIF) [file pone.0035911.s004.tif]

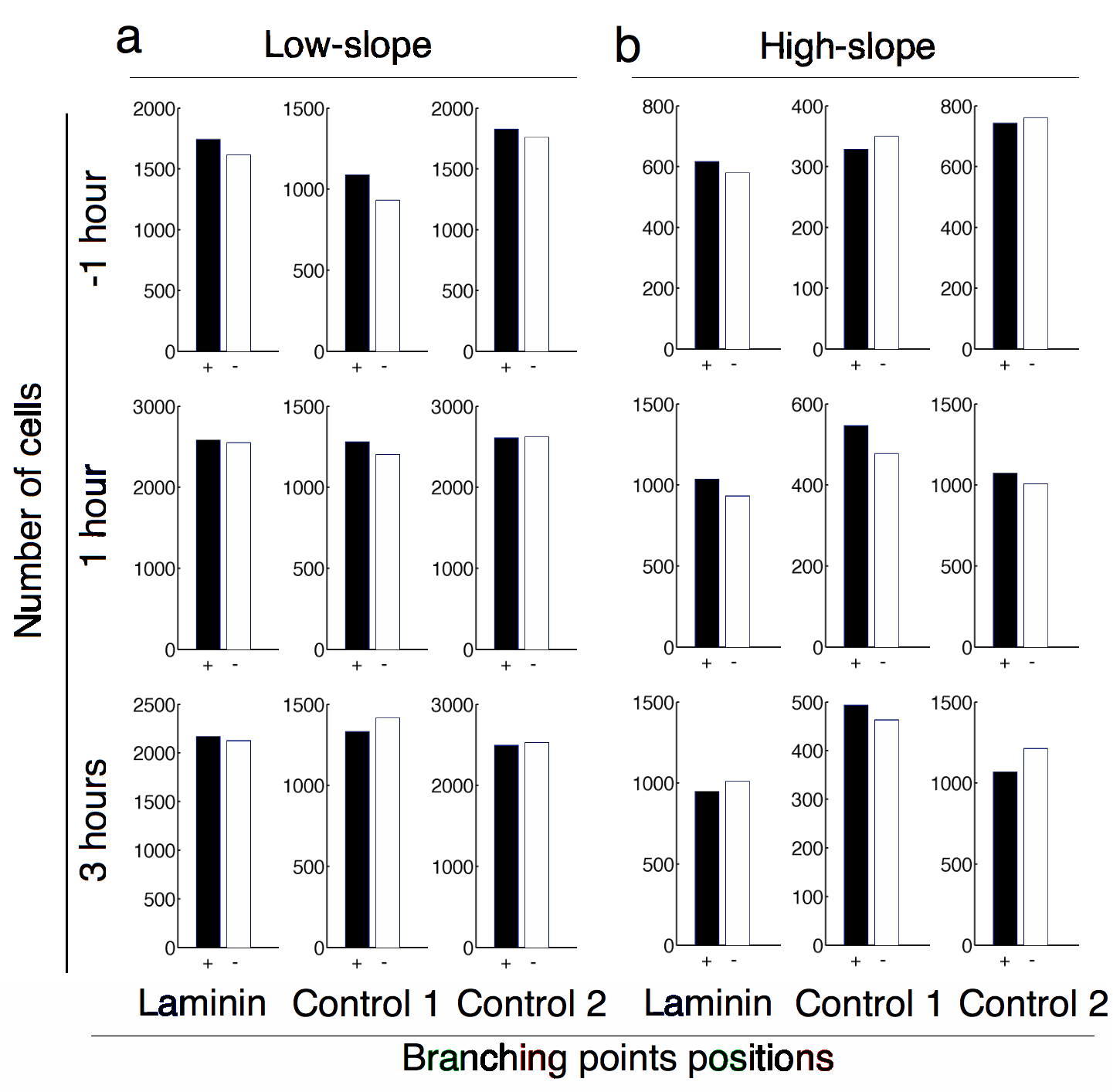

Supplement: Figure S5 — Histograms of the branching points of cells imaged on low-slope and high-slope gradients at different time points before and during differentiation by staurosporine. Attracted (black, +) and repulsed (white, −) neurites were determined as described in Figure 2a. (TIF) [file pone.0035911.s005.tif]

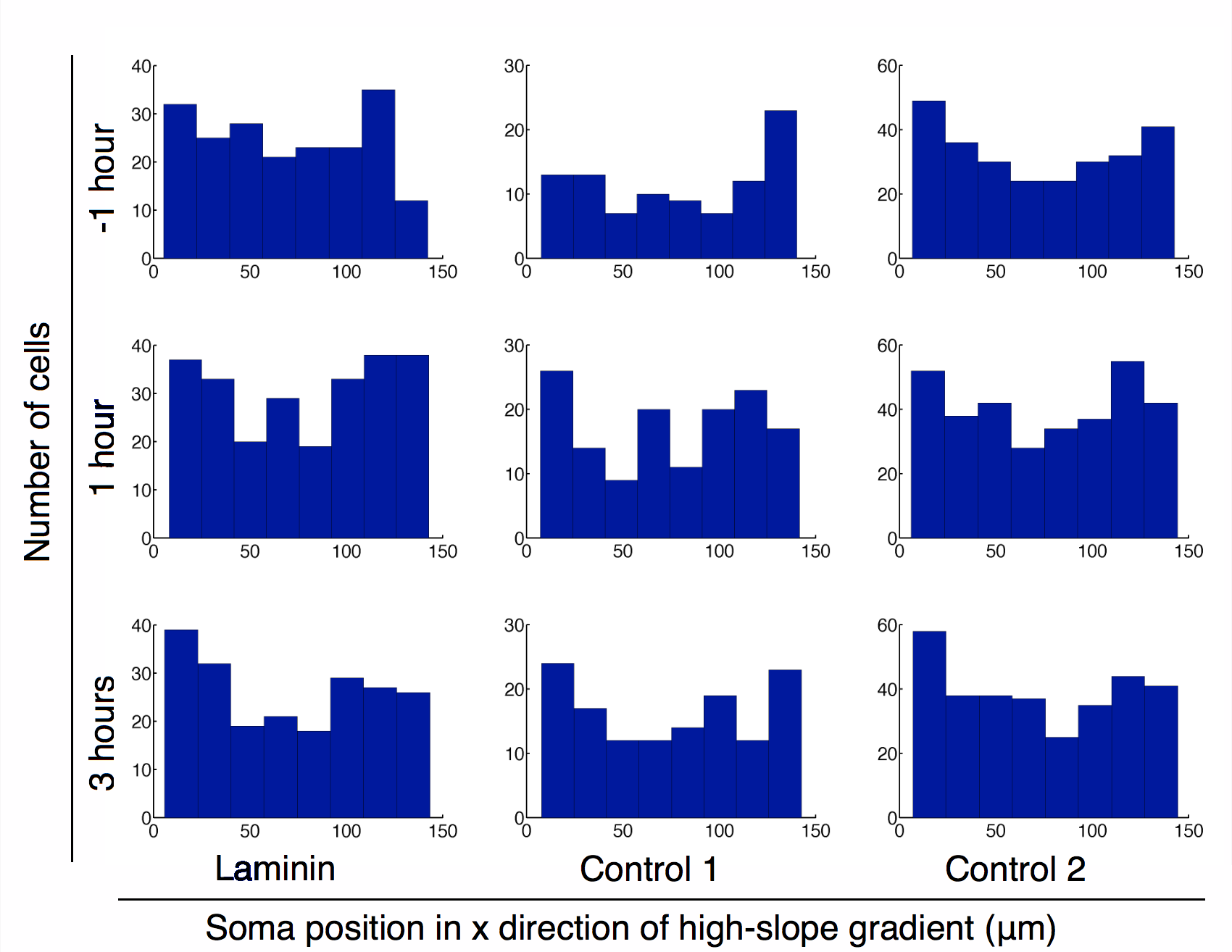

Supplement: Figure S6 — Histograms of the x-position of the centroid of the cell soma along the gradients (high-slope gradient). The laminin-1 or anti-laminin (control 1) concentration was highest at 0 µm and lowest at 148 µm. (TIF) [file pone.0035911.s006.tif]
